# Designing an mHealth Intervention for People With Visible Differences Based on Acceptance and Commitment Therapy: Participatory Study Gaining Stakeholders’ Input

**DOI:** 10.2196/26355

**Published:** 2021-03-24

**Authors:** Fabio Zucchelli, Olivia Donnelly, Emma Rush, Harriet Smith, Heidi Williamson, The VTCT Foundation Research Team

**Affiliations:** 1 The Centre for Appearance Research University of the West of England Bristol United Kingdom; 2 The Outlook Service Southmead Hospital North Bristol NHS Trust Bristol United Kingdom; 3 Vitiligo Support UK London United Kingdom; 4 see Acknowledgments

**Keywords:** mobile health, acceptance and commitment therapy, appearance, qualitative, participatory design, mobile phone

## Abstract

**Background:**

Given their growing popularity, mobile health (mHealth) apps may offer a viable method of delivering psychological interventions for people with an atypical appearance (ie, visible difference) who struggle with appearance-related distress. Acceptance and Commitment Therapy (ACT), a third-wave cognitive behavioral approach, has been used effectively in mHealth and is being increasingly applied clinically to common psychosocial difficulties associated with visible differences. We planned to design an ACT-based mHealth intervention (ACT It Out) for this population.

**Objective:**

The aim of this study is to gain key stakeholder input from user representatives and psychological clinicians to optimize the intervention’s design for future development and uptake. To do so, we explored considerations relating to mHealth as a delivery platform for adults with visible differences and elicited stakeholders’ design preferences and ideas based on initial author-created content.

**Methods:**

Within a participatory design framework, we used a mix of qualitative methods, including usability sessions and a focus group in a face-to-face workshop, and interviews and textual feedback collected remotely, all analyzed using template analysis. A total of 6 user representatives and 8 clinicians were recruited for this study.

**Results:**

Our findings suggest that there are likely to be strengths and challenges of mHealth as an intervention platform for the study population, with key concerns being user safeguarding and program adherence. Participants expressed design preferences toward relatable human content, interactive and actionable features, flexibility of use, accessibility, and engaging content.

**Conclusions:**

The findings offer valuable design directions for ACT It Out and related interventions, emphasizing the need to carefully guide users through the intervention while acknowledging the limited time and space that mHealth affords.

## Introduction

### Background

There are multiple reasons why someone may have an unusual physical appearance, or *visible difference*. Some live with a visible difference from birth, such as people with congenital craniofacial conditions, whereas others acquire a difference as a result of skin disease, injury, and/or medical treatment. In the United Kingdom alone, for example, around 1 in 60 people are estimated to have a visible difference [1]. Many affected individuals thrive; however, in appearance-focused Western cultures in which intrusive scrutiny of those who look different is commonplace, many others experience difficulties, including social anxiety and withdrawal, depression, body dissatisfaction, and low quality of life [2]. Specialist cognitive behavioral interventions show promise in addressing these concerns, typically incorporating social skills training to manage difficult interpersonal interactions [3,4].

In the context of limited specialist face-to-face psychological services for adults with visible differences internationally [5], there is an established need for self-help interventions catering to the specific experiences of this population [6]. Some prefer remote support, for example, because it is less stigmatizing [7]; others have limited or no access to specialist face-to-face services [8]. The review by Muftin and Thompson [8] provides preliminary support for web-based self-help in addressing appearance-based anxiety in adults with visible differences. However, the self-management landscape has since shifted, smartphones having overtaken laptops as many people’s primary electronic device [9], and individuals increasingly seek mobile health (mHealth) and mental health apps over web-based formats [10,11]. The aim of our overall project is to design, develop, and evaluate a standalone mHealth intervention, *ACT It Out*, for adults with visible differences experiencing appearance-related distress; to the authors’ knowledge, this is the first of its kind. This study describes formal stakeholder involvement at the design stage.

### Acceptance and Commitment Therapy

Acceptance and Commitment Therapy (ACT), an established third-wave cognitive behavioral therapy and behavior change model [12], underpins ACT It Out. ACT has been applied to mHealth interventions, with evidence for ACT-based mHealth in enhancing well-being and valued action [13], reducing social anxiety in a clinical population (alongside internet-delivered treatment) [14], and enabling smoking cessation [15]. Psychologists across Europe report using ACT with patients who have visible differences and note its suitability for the population [16,17]. A detailed exposition of how ACT fits the population’s needs is given by Zucchelli et al [18].

The process of change targeted in ACT is psychological flexibility, the capacity to direct one’s behaviors in accordance with personally held values, thus paying mindful attention both to facilitate valued action and to fully experience its fruition [19,20]. A total of 6 subprocesses mutually develop psychological flexibility: acceptance (willingness to experience private events including unwanted ones); cognitive defusion (loosening thoughts’ literality); present-focused attention, self as context (deidentifying from private events); understanding and clarifying one’s values (desired qualities of behavior); and committed value-oriented action [19]. Self-compassion is increasingly recognized as an inherent component of psychological flexibility and is specifically nurtured alongside the 6 subprocesses [21]. The converse of 2 of these subprocesses, cognitive fusion (converse of defusion) and experiential avoidance (converse of acceptance), have been shown to partially mediate the relationship between how people with visible differences evaluate their appearance and unhelpful appearance-focused behaviors, including avoidance of appearance-related situations and appearance-fixing behaviors such as covering areas of difference [22].

Psychological flexibility is promoted via mindfulness practices, experiential exercises, and metaphors, such as *passengers on a bus* [23]. This established metaphor describes how private events (thoughts, feelings, etc)—*passengers*—often seem to drive our lives. The metaphor invites us to imagine an alternative where we, the bus driver, take command of our direction of travel by establishing where we want to drive (via values) and by adopting an open, present, and detached relationship with the passengers and their protestations to accommodate their presence along the ride. The behavioral goal of ACT It Out is, therefore, to help users commit to more valued actions and engage in fewer avoidance-oriented behaviors (eg, avoiding social situations that evoke appearance anxiety).

### Stakeholder Involvement

ACT It Out is a complex mHealth intervention, namely, one with multiple interacting components targeting new behaviors from its users [24]. In the United Kingdom, where this project is based, the UK Medical Research Council recommends involvement from key stakeholders, including end users, at the design stage of complex interventions [25]. Participatory design methods, which facilitate representative end users’ perspectives, preferences, and ideas, are also vital in making any digital intervention appealing and accessible to its target user group [26]. Participatory methods and input from clinicians are also specifically recommended in mental health apps internationally to confer trustworthiness [27]. Accordingly, the authors collaborated in a participatory approach with experts by lived experience (*user representatives*) and clinical experience (*clinicians*) in designing ACT It Out. The aim of this study is to gain user representatives’ and clinicians’ perspectives using a participatory action procedure [28] to (1) explore the considerations relating to mHealth as a platform for delivering psychological intervention to the target group and (2) understand stakeholders’ design preferences and elicit design ideas based on viewing an initial version of ACT It Out created by the authors to help shape the design of ACT It Out. The technical and financial aspects of development are beyond the scope of this study.

## Methods

### ACT It Out Content

#### History of the Initial Design

In February 2018, we formed a project team of appearance psychology researchers, a lead clinician with extensive knowledge of ACT and visible difference, a lead user representative also with experience of running a vitiligo support charity (all of whom are coauthors), a human-computer interaction expert, and an app developer.

The researchers and lead clinician first sketched out a preliminary overview of ACT It Out, drawing from knowledge of ACT (including clinical experience in the case of the lead clinician) and self-help development as well as literature on ACT-based mHealth [29] and existing web-based programs for adults with visible differences (eg, Face IT) [30]. With input from the app developer, the project team created a mock-up of a small portion of the intervention. This was presented to members of organizations who represent a range of appearance-affecting conditions at a meeting in February 2018, to gauge interest, elicit ideas, and gain early feedback on design ideas. This study was undertaken before the formal process of stakeholder involvement, which forms the subject of this study.

#### The Design Presented to Stakeholders

**Figure 1:**
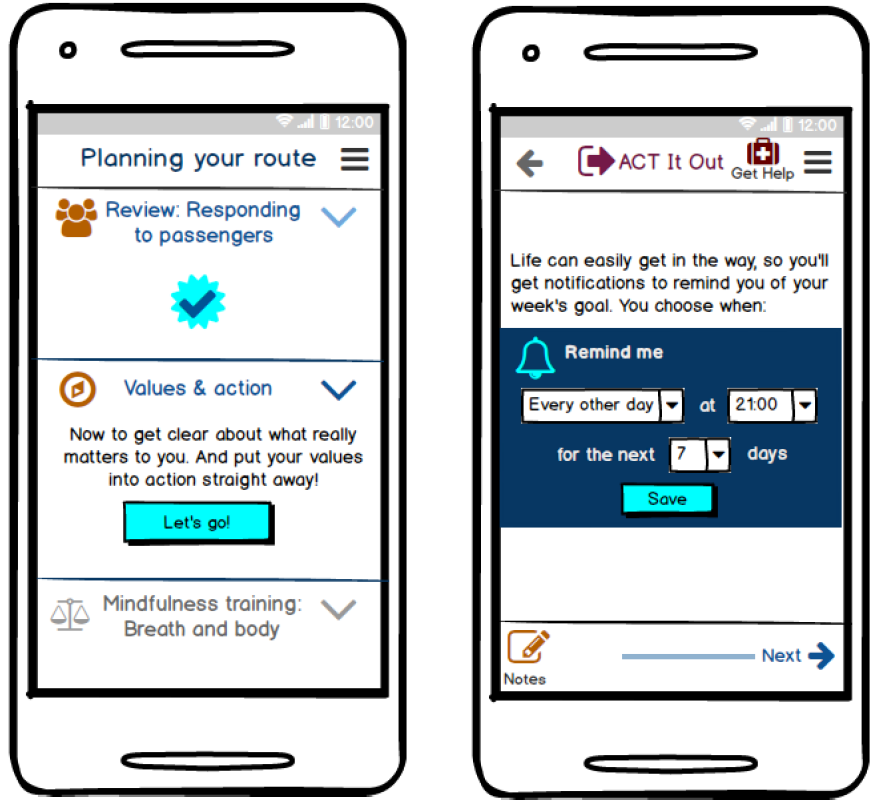
Example wireframe screens produced using Balsamiq.

In April 2018, we created a wireframe (screen-by-screen interface illustration) of the first 2 (of 4) sessions of ACT It Out using the software tool Balsamiq, based on the aforementioned background knowledge of authors and relevant visible differences, mHealth and ACT research, as well as feedback of organization members from the meeting in February 2018 (a wireframe screen is shown in Figure 1).

The wireframe designs subsequently presented to both stakeholder groups comprised 4 sequential training sessions of approximately 40 minutes, each subdivided into 3 subsections (Table 1). We envisaged that users would spend 1 week per session, during which they would engage in brief activities designed to cultivate psychological flexibility, such as guided mindfulness practices and carrying out self-set valued actions, aided by reminder notifications.

ACT It Out is facilitated by a preprogrammed human *guide* (an expert clinician) who appears in introductory videos in each session and is shown photographically offering textual tips and guidance throughout. The ACT model is introduced with an animated video used in previous ACT self-help interventions [29] showing *passengers on a bus*, which continues as the central metaphor throughout the sessions (eg, with users recognizing their common appearance-focused thoughts as *Your Appearance Passengers* in session 2). The way in which the ACT model relates to the common challenges experienced by people with visible differences is incorporated into the guidance and through real visible difference case examples. Bespoke guided mindfulness practices target acceptance, present attention, cognitive defusion, and self as context (*Attention on your 5 senses* in session 1, *Attention training: Breath and body* in session 2, *Attention training: Managing distress* in session 3, and *Attention training: In daily life* in session 4), alongside specific experiential cognitive defusion and self-compassion exercises. A modified values-sorting exercise [23] helps users clarify their values (*Your values in action* in session 3), and users progressively set short-, medium-, and long-term value-based goals. Social skills training, an evidence-based approach for adults with visible differences [3,4], is also presented as a contextually significant facilitator of behavior change toward valued action (*Building on your social skills* in session 3).

**Table 1:** High-level layout of the ACT It Out version presented to stakeholders.

Session and subsection		Main ACT^a^ processes targeted
**Introduction: Getting started**		
	Why ACT It Out?	N/A^b^
	How your data will be used	N/A
	Getting to know you	N/A
**1. Building awareness**		
	Passengers on a bus	Open up^c^
	Your appearance passengers	Open up
	Attention on your 5 senses	Be present^d^
**2. Planning your route**		
	Attention training: Breath and body	Self-compassion
	Recap and review of between-session activities	Valued action^e^
	Your values in action	Open up, be present
**3. Getting social**		
	Recap and review of between-session activities	Self-compassion
	Attention training: Managing distress	Open up, be present
	Building on your social skills	Valued action
**4. Putting your training into action**		
	Recap and review of between-session activities	Self-compassion
	Attention training: In daily life	Open up, be present
	Optional section: Intimacy and visible difference	Valued action
	Making a long-term action plan	Valued action

^a^ACT: Acceptance and Commitment Therapy.

^b^N/A: not applicable, as the introductory session does not target ACT processes.

^c^Open up: acceptance and cognitive defusion.

^d^Be present: present attention and self as context.

^e^Valued action: values clarification and committed action [23].

### Participants

The participants were 14 stakeholders: 6 user representatives and 8 clinicians. The authors recruited user representatives through purposively selected charities that serve a cross-section of appearance-affecting conditions, including cleft lip and/or palate, alopecia, burns, neurofibromatosis, vitiligo, and Apert syndrome. We purposively recruited for a gender mix (2 males) in user representatives and a wide age range (25-68 years), in an effort not to disadvantage older potential users. Eligibility included age over 18 years, self-identifying as having a visible difference, and some experience of psychosocial challenges relating to appearance. In addition to the 6 user participants, 1 individual the first author approached was unavailable, and 2 people agreed to take part but were later unable to attend because of personal reasons.

Through the authors’ professional networks, we purposively recruited specialist psychological clinicians who have experience of supporting individuals with visible differences and/or applying ACT in a health setting. Most were clinical psychologists (n=6) working for the UK National Health Service or support charities. In total, 4 clinicians were male and 4 were female. Another clinician the authors approached was unavailable to participate. All participants were White and were based in the United Kingdom.

### Design

#### Overview

**Figure 2:**
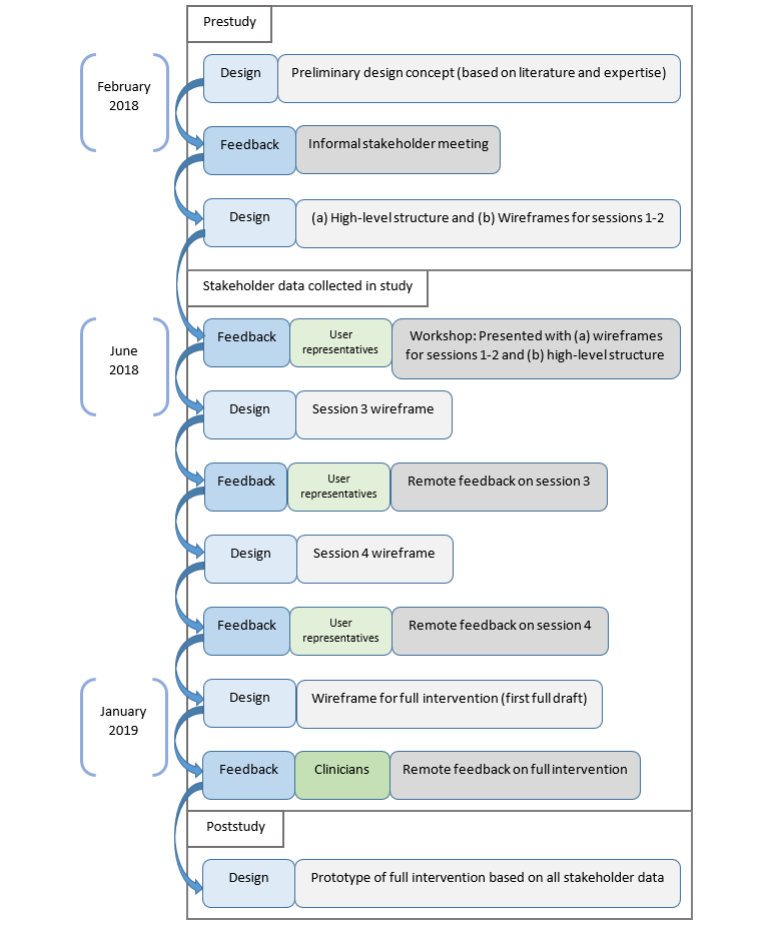
Diagram of the iterative design process used in ACT It Out. ACT: Acceptance and Commitment Therapy.

We employed participatory design methods to create a design via a series of iterations based on stakeholder contributions, as recommended for complex interventions and mHealth [25,27]. The authors front-loaded user representative input to ensure that the user perspective was incorporated into the first full draft of the ACT It Out design, before seeking clinicians’ input. The full iterative design process within which this study is conducted is shown in Figure 2. Ethical approval for this research was obtained from the University Research Ethics Committee.

#### User Representatives

We arranged a user representative workshop in June 2018. The authors chose a group workshop so that participants could meet each other, conducive to a sense of commonality, and to mitigate any potential power imbalance between participants and researchers. A total of 7 people accepted invitation and 4 attended (all 3 nonattendees notified the researchers).

The first author began the workshop by welcoming user representatives and giving an overview of the project, and then, the second author facilitated an *icebreaker* exercise. This was followed by one-to-one usability sessions, facilitated by trained researchers, of the ACT It Out introduction and sessions 1 and 2, in which user representatives viewed paper wireframes (following feedback from the February 2018 meeting that a nonclickable smartphone mock-up can cause frustration). Participants were invited to complete study-specific usability feedback forms and describe aloud their experience of using it as they progressed (*think-aloud* protocol) [31]. After a break, the participants took part in a semistructured focus group facilitated by the first and fifth authors. Topics included (1) advantages and disadvantages of mHealth as a self-help platform for individuals struggling with visible differences, (2) preferences for using mHealth (eg, duration and setting), (3) feedback on the high-level ACT It Out structure, (4) preferences regarding screen interface, and (5) feedback on specific design elements of ACT It Out. The focus group lasted for 1 hour and 39 minutes.

Following the workshop, one nonattendee viewed the introduction and sessions 1 and 2 wireframes remotely and completed the usability feedback form electronically. From the combined feedback, the first author produced a wireframe of session 3, then emailed this as a PDF file to user representatives to view independently and complete a feedback form. The first author then repeated this process in designing and gaining feedback for session 4, which 4 participants completed. In both stages, 4 participants took part. One additional workshop nonattendee viewed the entire wireframe remotely and provided written feedback. In total, 6 user representatives contributed to the design phase. The authors then discussed key design changes, and the first author incorporated user representatives’ feedback into the full intervention wireframes.

#### Clinicians

We then obtained feedback from expert clinicians from January 2019 onward. Due to busy work commitments of clinicians, the first author sent clinicians the wireframes as PDF files to view in their own time and asked them to complete a feedback form. The first author also arranged a telephone interview within a week of viewing the wireframes. Interviews were semistructured, following a largely equivalent schedule to the users’ focus group, along with more clinically oriented topics, including (1) defining the user group for whom ACT It Out is suitable (and unsuitable) and (2) design elements for behavior change. Interviews lasted for 43 minutes to 67 minutes (mean 51.63, SD 7.32).

### Data Analysis

The first and fourth authors pseudonymized and transcribed verbatim data from the user representative focus group, usability sessions, and clinician interviews. Together with written feedback, all data were transferred into NVivo version 12 for analysis. Data were analyzed using template analysis, an iterative form of thematic analysis in which a coding template is developed and modified throughout the analysis [32]. The authors chose template analysis for its compatibility with the project’s iterative, action-research paradigm and its accommodation of combined deductive and inductive analytical approaches. We deductively applied a priori themes from relevant mHealth literature (Textbox 1) to the first template, which were either removed or modified in light of new data. The authors also applied an inductive approach to capture any novel insights. The first author applied the first template to further data and incrementally refined the template where new data did not fit existing themes, culminating after 8 versions in a final template that encompassed all relevant data, on which the below results are based. The fifth author reviewed the analysis by checking transcripts and templates, resulting in the subsequent addition of the final (eighth) theme presented below.

**A priori themes from a literature review.**

A priori themes kept in the final coding template (although the wording was usually modified to better reflect participants’ accounts).

**Considerations of mobile health as a platform**

- **Themes retained**
  - Mobile health can augment therapy [34]
  - Ease of access and portability [28]
  - High dropout in mobile health [47]
  - Fear of iatrogenic affects [10]
- **Themes not retained**
  - Users’ data privacy [10,34,35,47]
  - Trustworthiness or credibility [11,27,35,47]

**Design preferences**

- **Themes retained**
  - Real-time engagement [33]
  - Reminders and notifications to engage [28,33,34]
  - Links to crisis support [27,33,47]
  - Immediate feedback [28,34]
  - Adaptable functionalities (eg, font and layout) [27]
- **Themes not retained**
  - Skepticism toward gamification [28]
  - Concise content [28]
  - Clear instructions for use [27]

## Results

### Overview

We produced 7 first-order themes, each split into lower-level themes, and 1 integrative theme (full details of the themes, their relation to the research questions, and which participant groups contributed toward each theme are given in Multimedia Appendix 1). The central findings from the combined participant groups, with illustrative quotes, are provided in the following sections. To preserve anonymity, participants’ comments were labeled by the stakeholder group only.

### 1. mHealth Has Advantages for Users

Participants expressed the advantages of mHealth interventions over traditional talking therapies and other self-management platforms. Participants valued the privacy afforded by mHealth in terms of its discretion and suitability for those preferring not to seek face-to-face support for appearance concerns:

[An app] is perfect for someone who doesn’t want to go and get counselling, this is a self-help thing that we all do and it’s very modern so it’s something that is needed...plus reading it on an app, it’s not like you’re reading a self-help book and everybody else can see what you’re reading; it’s personal.

User representative, focus group

Participants’ accounts also highlighted the autonomy offered by mHealth through its portability and accessibility:

I like apps as an idea because you can use them wherever you are. Suppose you’ve got your meditation tabs [on the app]...Going to the pub and having a difficult moment, it’s good to be able to access it there and then.

Clinician, interview

### 2. mHealth Should Add to—Not Replace—Existing Face-to-Face Resources

Participants felt that mHealth could complement existing face-to-face psychological support before, during, or after face-to-face support:

[mHealth] might be a step toward thinking about what help [users] might need. So it could be a platform for other things...I got excited [looking at the wireframe]; I could use this alongside working with somebody. You could alternate them doing something and then having a conversation about it.

Clinician, interview

Clinicians, in particular, voiced their view that mHealth cannot and should not replace face-to-face support:

As an adjunct, or something that’s available where nothing else is available I think [mHealth] has potentially a huge benefit─but I’d not want to see it as a replacement for all individual therapy.

Clinician, interview

Relatedly, participants suggested that mHealth may be less suitable for some, including individuals with high levels of need or recently acquired visible differences:

[An app is] possibly [not suitable for] people who have only acquired a visible difference recently, because they may not know they have trauma responses, or they may need more time before they jump into something like this.

Clinician, interview

### 3. Safeguard Users’ Well-Being

Participants emphasized the need to embed content and features that safeguard users’ well-being:

...have a section [in the app] with useful links so you could fast-track to all of the different links and organisations where you can go [for support].

User representative, focus group

Is there a fail-safe mechanism, if someone has checked too hard all the way through [in response to questions asking if users would like to work on specific social skills]─have a screen that pops up and checks in with them.

User representative, written feedback

Possible discomfort is part of the process [of ACT], and [users] need to know that before [they] commit to it. And you might decide that if you’ve got particular things going on in your life, “You know what, not right now,” or “Now is exactly the time I want to do this.” But doing it knowing this isn’t going to be a series of aromatherapy massages where at the end of each one you’ll expect to feel much nicer than when you started.

Clinician, interview

### 4. There Has to be That Human Link

Participants described the importance of ACT It Out establishing a human connection with users. Many preferred the idea of a single preprogrammed human *guide* with relevant experience over multiple guides:

I think there’s a lot to be said about there being a teacher. Certainly within the world of mindfulness, there’s a healthy attachment to a teacher figure...You want a face to go with the voice. There has to be that human link to get people going.

Clinician, interview

User representatives, in particular, felt that the in-built human guide needs to be responsive to user input, to validate the common experiences of people with visible differences and offer encouragement:

Just have options with a dropdown menu, [eg,] If [a task] made me feel [unconfident], and then you give a bit of advice like “It’s ok to feel like this, it’s ok that you feel a little bit less confident right now.”

User representative, focus group

...if there was some feedback loop in there, [users] might be more inclined to put something in.

User representative, focus group

Participants from both groups highlighted the need for ACT It Out to offer a way of normalizing users’ experiences and saw real-life case examples as the best way of doing so, while also helping users to buy into the intervention’s processes:

...the [real] examples are very good, [to see] that’s how other people cope and see real experiences not just what experts think. It has to come from an actual person.

User representative, usability session

### 5. Engender Action

Participants were clear that a core design priority of the app should be to elicit value-based behavior change. To do so, participants suggested that content should focus on actionable tasks:

I felt like one really strong bit is the social skills bit. Because it was really practical, gives some clear guidance.

Clinician, written feedback

User-set notification reminders for activities were seen as a crucial tool unique to mHealth to aid engagement with behavioral tasks:

User representative (usability session): Would you be able to choose when you get your notifications?

Researcher: Yeah, does that feel important to you?

User representative: Yeah

I love the capacity to have reminders and the user to have control around the timings etc.

Clinician, written feedback

Clinicians described the importance of ACT It Out facilitating both immediate and sustainable behavior change, for example, by providing sufficient time between sessions for practice and by breaking behavioral goals into discrete, manageable segments:

Selecting your values and then implementing them as short, medium and long-term goals. I could see the benefit of that.

Clinician, interview

### 6. Design for a Range of Users

Participants’ divergent app use preferences highlighted the need to build in flexibility about how the program is used:

It’s helpful to have a journal and plus at the end of it you can always go back to see how far you’ve come. I don’t want to sit there and write down how I’m feeling.

User representative, focus group

My train journey from where I am to go anywhere is 45 minutes and [I] sit on a different app for the full 45 minutes, so that doesn’t seem very long to me [to use an app].

User representative, focus group

I just think it’s kind of human nature, we don’t want to sit down and spend a lot of time on [an app].

User representative, focus group

With ACT It Out designed for people with a range of causes of visible difference, participants noted the physical usability issues that may exist for some and the need to design the screen interface accordingly:

As well as facial differences, a lot of people with burns or other conditions might not have [fully functional] fingers so it’s being able to press something...I found it yesterday logging onto [a public transport app], a tiny tick-box for terms and conditions, I couldn’t press it because the button was far too small for my finger...it would have to be a nice big button.

User representative, usability session

### 7. Design for Learning

Participants’ feedback also suggested that the content and structure of ACT It Out should actively facilitate user learning. Participants sought a clear rationale for each element of training:

Can you say something more here [in the first mindfulness training section] about mindfulness? Why is mindfulness helpful? Why are we asking people to do it?

Clinician, written feedback

Participants expressed a desire for training content to be organized into short, sequential chunks that consolidate and build on preceding sessions:

I like that it’s broken down into different sections...and also within each session I think is helpful. Because psychological information can be a bit jargon-heavy, a bit much.

Clinician, interview

I can imagine that people going through it maybe have a tendency to go “I know what that’s all about” and move on, but it looks like you’ve built it in so you’d have to actively click Next. I think the way it’s laid out is quite easy to follow and gradually builds up.

User representative, focus group

Clinicians felt that learning would be aided by linking all training material to a small number of simple, actionable models:

I think having the Choice Point [a visual ACT model] in each session would be a really good way of tying the content together. Something quite visual...if [a user] is new to [ACT], just having one or two things that we just keep coming back to again and again.

Clinician, interview

### 8. Mitigate Dropout

An integrative theme that permeated across themes 3 to 7 conveyed participants’ concern over users discontinuing the use of ACT It Out:

I suppose the fear would be someone starts and then doesn’t carry on...before they get to the good bit.

User representative, focus group

...if somebody comes into [ACT It Out] with high expectations but significant problems, are you actually going to add to the problem because they’re going to fail through the app? And how to manage that process?

Clinician, interview

## Discussion

### Principal Findings

In this study, we aim to identify the considerations of mHealth as a therapeutic platform for adults with visible differences and the design preferences and ideas of key stakeholders to optimize the design of ACT It Out. The 8 themes subsequently informed the full redesign of ACT It Out to be piloted in the prototype form.

Participants’ accounts highlighted strengths, challenges, and limitations of mHealth as a mode of delivering psychological interventions for adults with visible differences. The strengths expressed by participants, namely, user discretion, accessibility, and portability, have been reported in the mHealth literature [33,34]. These features are likely to be of particular use to those experiencing social anxiety, a common challenge for people with visible differences [2,36-38]. Using a mobile platform and aided by social skills training, value clarification, and behavioral action plans, ACT It Out may offer a suitable and accessible medium for users to potentially transition into greater social activity.

In terms of challenges, participants clearly expressed the need to safeguard users’ well-being and mitigate potentially deleterious effects. The potential for iatrogenic effects has previously been highlighted as a concern for mental health apps [10]. Within the training content, clinicians, in particular, highlighted the need to provide clear, regular, and timely information on the ACT processes underpinning ACT It Out to manage users’ expectations and offer informed consent on its ongoing use. Although the effectiveness of trigger warnings before potentially distressing material and exercises remains unsubstantiated [39], providing users with relevant information about all exercises in advance and treating informed consent as an ongoing process is in keeping with ethical guidelines for psychological intervention more broadly [40].

Participants’ feedback suggests that the absence of live interaction with a real therapist may pose an advantage for those who prefer remote support and a limitation for others. Establishing a *human* element to ACT It Out was, though, favored unanimously, corresponding with a growing interest in facilitating a therapeutic alliance in mHealth [41] and user preferences for embodied, empathic chatbot hosts rather than anonymous avatars [42]. Using a human *guide* via videos, photographs, and text, who responds to user input, was deemed crucial to providing meaningful interaction and validation of users’ experiences. Nevertheless, clinicians’ feedback highlighted that mHealth cannot fully replicate face-to-face support, and hence, ACT It Out would be unable to offer adequate support for those with greater clinical needs. Therefore, we need to provide clear guidance on who it is designed for and for whom professional support may be more suitable, both in promoting ACT It Out and in its content.

Various design preferences expressed by participants have since been incorporated into ACT It Out and may be informative for those developing any related interventions. Real case examples were resoundingly popular, offering a way of normalizing the typical difficulties experienced by people with visible differences, counteracting a felt sense of difference common to this group [2]. Such case examples may also confer credibility to the ACT approach. In keeping with the participatory action paradigm, some of the user representative participants offered their own stories for future iterations of ACT It Out.

Participants’ accounts pointed to the need to design content and features to encourage concrete behaviors. User-set notifications were valued by all participants as reminders for activities, echoing previous research on mental health apps [43]. Designing precise and time-limited value-based goals, with reminder notifications that appear on the same device through which many of the training activities are undertaken, offers a powerful tool for enacting implementation intentions (a strategy of specifying when and how an individual engages in goal-directed behavior) of the type targeted in ACT It Out [44]. As raised by clinicians, sufficient time is needed to establish and sustain behavioral changes, such as regular mindfulness and social skills training practice. To offer greater opportunity for behavior change and to reflect the mean duration spent by users on cognitive behavioral apps (5.4 weeks) [10], the authors have since increased the ACT It Out training content from 4 to 6 weekly sessions, without increasing the overall content.

Participants’ preference for building the training content in a systematic, step-by-step fashion with manageable amounts of information corresponds to the user experience principle of progressive disclosure, in which only information essential to any given step of a process is provided when users need it [45]. This is especially important in mHealth design, where there is less space and time to engage users’ attention [46]. Therefore, we simplified and divided the training content in accordance with this principle.

Participants’ feedback also highlighted that not all user design facets are universal, with users likely to vary in their interaction preferences. Therefore, we included an optional journal or notes feature throughout the training for those who wish to use it. The need to accommodate users’ varying physical needs is also paramount, especially for a user group in which many people have conditions that also affect physical function (eg, impaired digit functioning, hearing loss) and appearance. Features such as large font and buttons and optional subtitles and scripts for recordings offer the sort of adaptive functionality recommended in mHealth design [27].

The relatively high mHealth user dropout rate established in the literature [35,47] was echoed by participants’ concerns. Many of their design preferences sought to foster engagement and counter potential causes of attrition, such as overly challenging materials.

### Limitations

A limitation of the study was the small sample size, especially of the user representative group. The project was conducted primarily as stakeholder collaboration rather than in a traditional researcher-as-expert paradigm, as befits the development of a complex intervention [25]. This meant that the authors prioritized the quality of relationships with user representatives over their quantity. Collaborating with a small number of engaged user representatives over a year meant that the group was well informed about the project’s objectives and their role within it. A second limitation was that the first author and lead designer of ACT It Out was heavily involved in data collection, creating the possibility of acquiescence bias in participants’ responses [48]. The first authors’ unique knowledge of ACT It Out meant that he was nevertheless best placed to gain feedback from participants and could follow up on participants’ responses during data collection.

### Conclusions

By collaborating with key stakeholders, namely, user representatives with visible differences and clinicians, this study established several actionable directions for the mHealth intervention (ACT It Out) under development. Gaining both user and clinician perspectives gave us a comprehensive picture of what an mHealth intervention based on ACT should look like for the target population. This paper also offers an example for other researchers involved in developing mHealth and other complex behavioral interventions and allows the authors’ methods to be critiqued [25].

## Appendix

Multimedia Appendix 1 Themes mapped to research questions and their respective participant groups.

## Abbreviations

ACT: Acceptance and Commitment Therapy
mHealth: mobile health
