# Supplementary material for: Designing an mHealth Intervention for People With Visible Differences Based on Acceptance and Commitment Therapy: Participatory Study Gaining Stakeholders’ Input
Source: JMIR Form Res. 2021 Mar 24;5(3):e26355. doi: 10.2196/26355 (PMC8075076; doi:10.2196/26355)
Supplement: Multimedia Appendix 1 [file formative_v5i3e26355_app1.docx]

| Relevant research question | First-order theme | Second-order themes | Third-order themes | Integrative theme |
| --- | --- | --- | --- | --- |
| Considerations of mHealth as a platform | Advantages of mHealth for people with visible differences | Privacy of mHealth^a^ | mHealth is discrete^a^  MHealth suits those not wanting face-to-face^a^ |  |
|  |  | Autonomy^a^ | mHealth can be used in own time unlike therapy  Portability & accessibility^a^ |  |
|  |  | mHealth has wide reach^1^ |  |  |
|  | mHealth should add to- not replace- existing face-to-face interventions | mHealth can complement face-to-face^a^ | mHealth as a stepping stone to health services  Useful as adjunct to / while waiting for face-to-face^a^ |  |
|  |  | mHealth can’t replace face-to-face^1^ | mHealth not suitable for everyone (higher need, PTSD etc.)^1^  Harder to manage risk/expectations in mHealth vs face-to-face^1^ |  |
|  |  | mHealth good in absence of specialist support^1^ |  |  |
| Both research questions | Safeguard users' wellbeing^a^ | Easily accessible links to support services^a^ |  | Mitigate dropout^a^ |
|  |  | Responsiveness to signs of difficulty |  |  |
|  |  | Inform users about ACT processes^1^ |  |  |
|  | “There has to be that human link” | Humanised interactivity stimulates engagement | Encourage user response  Responsiveness from the app validates users’ experience |  |
|  |  | Real examples normalise users’ experiences |  |  |
|  |  | One human app guide throughout |  |  |
| Design preferences | Engender action | Make behaviour change achievable^1^ | Break down goals clearly^1^  Sufficient time to adopt and sustain new behaviours^1^ |  |
|  |  | Actionable tasks^a^ |  |  |
|  |  | User-set notifications aid action^a^ |  |  |
|  | Design for a range of users^a^ | Flexibility for divergent user preferences^2,a^ | Offer different input methods^2, a^  Time spent per use likely to vary^2^ |  |
|  |  | Account for differing needs of group^a^ | Screen interface – consider needs of group^a^  Acknowledge variation in users’ social functioning^1^ |  |
|  | Design for learning | Giving rationale for content aids user buy-in |  |  |
|  |  | Tie all content to simple, repeating models | Align social skills training to ACT model  Offer various simple techniques and metaphors^1^ |  |
|  |  | Build in blocks | Sequential structure keeps users on track  Break down ACT It Out into clear, small chunks  Consolidate learning throughout |  |

^1^ = Themes developed from clinician data only, ^2^ = Themes developed from user representative data only. All other themes include both. ^a^ = A priori (deductive) themes.
